# Diclofenac Sodium for Fever Control in Neurocritical Care: A Systematic Review

**DOI:** 10.3390/jcm12103443

**Published:** 2023-05-13

**Authors:** Tommaso Rochat Negro, Michael Watchi, Hannah Wozniak, Jerome Pugin, Herve Quintard

**Affiliations:** 1 Intensive Care, Hôpitaux Universitaires de Genève, 1205 Genève, Switzerland; hannah.wozniak@hcuge.ch (H.W.); jerome.pugin@hcuge.ch (J.P.); herve.quintard@hcuge.ch (H.Q.); 2 Intensive Care, CHU de Poitiers, 86000 Poitiers, France; michael.watchi@chu-poitiers.fr

**Keywords:** diclofenac sodium, fever, acute brain injury, management

## Abstract

Background: Fever is extremely common in neurocritical care patients and is independently associated with a worse outcome. Non-steroidal anti-inflammatory drugs (NSAIDs) lower the hypothalamic set point temperature through the inhibition of prostaglandin E2 synthesis, and they constitute a second line of pharmacological treatment for temperature control. This systematic review aims to evaluate the effectiveness of DCF in reducing body temperature and its effects on brain parameters. Methods: A comprehensive search of several databases was run in November 2022 in Ovid EBM (Evidence Based Medicine) Reviews, Cochrane library, Ovid Medline and Scopus (1980 onward). The outcome of interest included DCF control of body temperature and its impact on cerebral parameters. Results: A total of 113 titles were identified as potentially relevant. Six articles met eligible criteria and were reviewed. DCF induce a reduction in body temperature (MD, 1.10 [0.72, 1.49], *p* < 0.00001), a slight decrease in ICP (MD, 2.22 [−0.25, 4.68] IC 95%; *p* < 0.08) as well as in CPP and MAP (MD, 5.58 [0.43, 10.74] IC 95%; *p* < 0.03). The significant heterogeneity and possibility of publication bias reduces the strength of the available evidence. Conclusions: Diclofenac sodium is effective in reducing body temperature in patients with brain injury, but data in the literature are scarce and further studies are needed to evaluate the benefits of DCF.

## 1. Introduction

Fever is routinely described as a core temperature above 38.5 °C, even if this threshold remains a nature of debate [1,2]. In neurocritical care, almost 70% of patients admitted following acute brain injury may present fever which is independently associated with a poor outcome [3,4]. Fever induces release of excitatory amino acids (glutamate and dopamine) and free radicals [5]. It also produces an ischemic depolarization and a breakdown on the blood–brain barrier which leads to the development of cerebral oedema [6]. Elevation of the brain temperature also correlates with an increase in cerebral oxygen consumption, brain metabolism, and intracranial pressure [7,8,9,10,11]. However, despite its major role, the definition of fever and its management are still a matter of debate.

Nowadays, fever in intensive care settings is commonly managed with a multistep approach [12]. It is first controlled by administration of antipyretic medications. Acetaminophen remains the first and most used agent. Non-pharmacological treatments are solely used when fever is refractory to the first line. Passive external cooling with a reduction in the air temperature of the room or by applying cold blankets and ice packs on the patient skin are then used. The last line of therapy consists of invasive measurement with placing the patient on a controlled and cold matrass or with the insertion of devices into the blood circulation (Artic Sun ^®^, Becton, San Jose, CA, USA; CoolGard ^®^, Sydney, Australia). Both passive external and invasive cooling can lead to discomfort, intolerance or shivering and require sedation of the patient.

Nonsteroidal anti-inflammatory drugs (NSAIDs) are part of the pharmacological therapy. They are known to lower the hypothalamic set point temperature through the inhibition of prostaglandin E2 synthesis, and they constitute a second line of pharmacological treatment for temperature control [5,13]. Yet, antipyretic drugs carry specific and predictable side effects. NSAIDs administration has been reported to cause hypotension, impaired hepatic and renal function, sodium and water retention, and severe oliguria [14,15,16]. There may be also a theoretical concern for their antiplatelet effects in patients who have bled intracranially for extending the hemorrhage. However, for diclofenac, these side effects appear to be present for repeated doses and above 150 mg/day [17].

Among NSAIDs, diclofenac sodium (DCF) is the second choice of pharmacological molecule in Europe for the treatment of fever in neurocritical care setting [1]. Despite its use is reported since the 1990s, fears concerning hemodynamic impact and infection make it not suitable as second-line pharmacological treatment. This systematic review aims to evaluate the effectiveness of DCF in reducing body temperature, its possible effects on brain parameters and its safety.

## 2. Materials and Methods

A systematic review was performed to evaluate the efficacy of DCF to control body temperature and its impact on cerebral parameters. The protocol was registered on the PROSPERO website (CRD42023359981). This article adhered to the latest Preferred Reporting Items for Systematic reviews and MetaAnalyses statement [18].

Eligible studies included original research evaluating the effect of DCF on body temperature management, changes in hemodynamic and brain parameters or reporting adverse events in acute brain injury patients, independently from the etiology and from the root of administration of DCF. Inclusion was restricted to either randomized controlled trials (RCTs) or observational studies.

To meet eligibility criteria, studies had to involve adult patients (age ≥ 14 years) with an acute neurologic emergency recovered in ICU and with the presence of fever treated with DCF. In addition, studies had to report at least one of the outcomes of interest mentioned above to be considered eligible. Eligibility was not restricted to the language of publication and no time limit was applied to the research. Case reports were not included.

### 2.1. Information Sources and Search Strategy

A comprehensive search of several scholarly electronic databases was run in November 2022 in Ovid EBM (Evidence Based Medicine) Reviews, Cochrane library, Ovid Medline and Scopus (1980 onward). Search filters were applied to remove most animal studies and case reports. Detailed search strategies are provided in Supplementary Materials. The list of references of eligible studies was also reviewed to avoid missing any important studies. Gray literature was not searched.

A pair of reviewers (TRN and MW) independently considered the potential eligibility of each title and abstract that resulted from executing the search strategy. Records considered potentially eligible were then assessed in full text for eligibility by two independent reviewers. Disagreements were resolved by consensus or by arbitration with a senior author (HQ).

### 2.2. Risk of Bias Assessment

Risk of bias was evaluated at the outcome level for each eligible study. For the only RCTs, the risk of bias was assessed by using version 2 of the Cochrane risk of bias tool (RoB 2) [19]. For observational studies, the modified Newcastle–Ottawa scale tool was used [20].

Table 1 resumes the methodological quality scores of each study with the five cohort studies obtaining a NOS above the quality threshold set at three stars on nine. According to the RoB 2 score, risk of bias in the Cormio et al.’s study of 2007 presents many concerns, especially regarding deviation from the intended interventions, measurement of the outcome and selection of the results.

All studies were assessed by two independent reviewers (TRN and MW), and disagreements were solved by discussion and, when not possible, with the help of a senior methodologist (HQ).

Data were pooled using a random-effect model, results were abstracted as standard mean (MD) and 95% confidence interval (CI). Heterogeneity was assessed by calculating chi-square (I^2^). To note, studies included in this meta-analysis present a high heterogeneity which is above 80%.

## 3. Results

Both reviewers screened a total of 113 titles and abstracts, among which 13 studies were identified as potentially relevant. After removing duplicated papers, six articles met eligible criteria and were reviewed [21,22,23,24,25,26,27]. Of the six articles, three were prospective studies, two were retrospective studies, and only one randomized controlled trial was found. The population studied in the articles was specified in five of them and consisted of patients admitted in neuro-ICU following subarachnoid hemorrhage (SAH), intracranial hemorrhage (ICH), trauma brain injury (TBI), acute stroke and immediate post-neurosurgery.

All the studies were focused on DCF use in fever management during the early phase of neuro-ICU admission. Two studies were built to demonstrate the efficacy of continuous intravenous infusion of low-dose DCF and its effects on cerebral and hemodynamic parameters; one study compared continuous low-dose infusion with intermittent bolus; one observed cerebral parameter effects of low-dose intravenous infusion of DCF and two studies were conducted to prove the efficacy of intramuscular and subcutaneous DCF in fever management and its hemodynamic effects.

**Figure 1:**
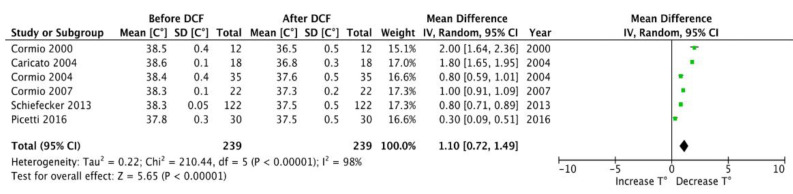
Forest plot: Impact of DCF on temperature [22,23,24,25,26,27].

**Figure 2:**
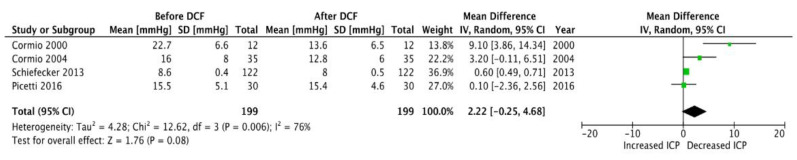
Forest plot: Impact of DCF on ICP [22,23,24,25,26,27].

**Figure 3:**
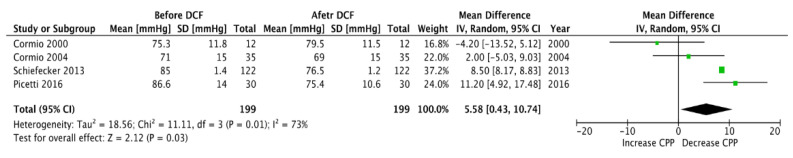
Forest plot: Impact of DCF on cerebral perfusion pressure (CCP) [22,23,24,25,26,27].

A total of 239 uses of DCF in the setting of fever management in neuro-ICU were reported in the six studies, for a total of 146 patients. In six studies, which count 239 reports, low dose of DCF administration was in favour of a better fever management with a decrease from 38.5 °C (±0.4 °C) to at least 37.6 °C (±0.5 °C) within a few hours of use (MD, 1.10 [0.72, 1.49] IC 95%; *p* < 0.00001) (Figure 1). The cerebral parameter effects of low-dose DCF were monitored and discussed in four of the six studies, which reported 199 uses on the 227. Overall, only a slight change in ICP was observed (MD, 2.22 [−0.25, 4.68] IC 95%; *p* < 0.08) (Figure 2). Instead, a decrease in CPP was observed after its administration (MD, 5.58 [0.43, 10.74] IC 95%; *p* < 0.03) (Figure 3).

In order to measure local changes in brain parameters, brain metabolism and brain oxygenation, Schiefecker [25] monitored the brain oxygenation saturation (PbtO2) of their patients (122 interventions) with the implantation of a probe. A 13% decrease in the mean PbtO2 of 28.1 ± 2.2 mmHg was observed and was associated with brain tissue hypoxia in 35% of the interventions. This decrease in PbtO2 was simultaneous to a decrease in CPP, although a direct effect of DCF on both parameters could not be identified.

During the administration time, patient systemic hemodynamic parameters, heart rate and mean arterial pressure were continuously monitored, respectively, in four and six studies. DCF administration had no impact on heart rate. A decrease in overall mean arterial pressure was recorded with a decrease of −8.65 mmHg (IC 95% [6.11, 11.2]; *p* < 0.00001) (Table S1, Supplementary Materials).

Adverse events consecutive to DCF administration such as GI bleeding, alterations of renal and liver functions were monitored in four studies (Table 2). In Cormio’s [22] study, liver function, kidney function and urinary output were monitored daily before and after DCF administration, with no significant changes. Similarly, in the other study by Cormio [21], in addition to the liver and kidney control with daily laboratory tests, occult blood tests in stomach and stools were performed with the aim of ruling out a possible increase in gastrointestinal bleeding. No significant difference or increase in complications after DCF administration was reported. In the last two studies (Cormio 2004; Caricato 2004) [23,24], it was reported that side effects were monitored and not observed, but without specifying methods and timing.

Infection screening was mentioned in four studies, but most of the patients included already received antibiotics. Specifically, in one study (Cormio 2000) [22], the start of DCF was reported while the infectious disease that was believed to be the cause of fever was already being treated. Pneumonia was diagnosed in 84% and sepsis in 16% of patients. Antimicrobial therapy was empiric or tailored to the known or likely aetiologic agent. All causes of fever in this study were reported to be of infectious origin. In the second study conducted by Cormio [24], infections were reported to be clinically suspected in all patients, and patients were treated with antibiotics. Of the total of 26 infections diagnosed (11 in the DCF group and 15 in the control group), the predominant site of infection was the lung: 19 patients had ventilator-associated pneumonia, two had meningitis, and four were affected by other infections. In the third study (Caricato 2004) [23], 14 of the 18 patients involved presented a fever of infectious origin with positive blood cultures; for the remaining four patients, the authors reported that the fever was of central origin.

Neurological follow-up was mentioned in two studies, one using the Modified Rankin Scale at 3 months and the second the Glasgow Outcome Scale score at 6 months. Both did not report any change in the neurological outcome, but the reduced number of patients makes a general extrapolation impossible.

## 4. Discussion

The presence of fever represents a secondary insult in brain injury patients, and brain temperature exceeding >37.5° correlates with increased ICP and altered CPP [28]. The results of this systematic review including 146 patients with 239 measurements show that the use of diclofenac sodium effectively reduces body temperature in neurocritical care patients. However, independently of the way of administration, DCF induces a slight decrease in ICP and a more pronounce decrease in CPP due to its hemodynamic effects which causes decrease in the MAP.

**Figure 4:**
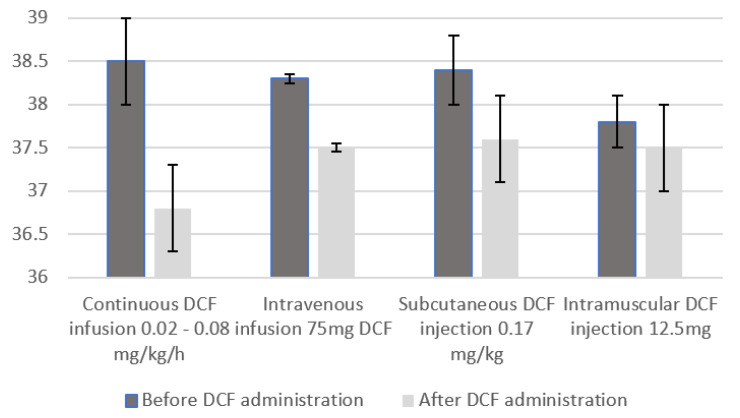
Body temperature changes after diclofenac administration according to its way of administration and its dose.

When analyzing the effect on temperature, and regardless of dosage and route of administration, which are extremely heterogeneous, a significant reduction in body temperature following DCF administration is reached (MD, 1.10 [0.72, 1.49], *p* < 0.00001) with a body temperature decreasing under the commonly used threshold of fever of 38.5 °C (reduction to 37.6 °C (±0.5 °C). This reduction seems to be greater when DCF administration is intravenous and continuous, thus not showing a linear relationship between dosage and temperature lowering (Figure 4). A hypothesis could be that low doses of DCF are probably sufficient to inhibit PGE2 production and lower the hypothalamic set point. However, current studies lack the power to prove the linearity of this relationship. Moreover, the anti-pyretic effect of DCF was homogeneous despite the variety of brain damage present in the different studies. In fact, five out of six studies presented patients with different pathologies (TBI; SAH; ICH).

**Figure 5:**
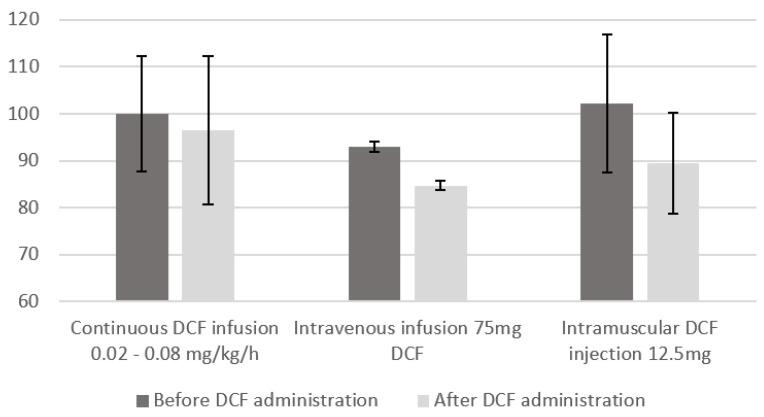
Impact of Diclofenac administration on mean arterial pressure (MAP) according to its way of administration and its dose. To note, no report of the impact on MAP of the subcutaneous infusion of DCF could be shown as date was not available.

We observed a hemodynamic effect of DCF with a reduction in MAP and CPP after its administration. However, this effect changes between studies and is greater in those that used higher doses of DCF, discontinuous and intravenous administration. This trend seems to encourage the use of diclofenac at low doses and in continuous infusion in order to avoid blood pressure fluctuations (Figure 5).

In this systematic review, we also investigated whether the use of DCF could have an impact on ICP. Not all studies reported ICP values, but from the data collected, the reduction in ICP values after DCF administration is minimal or null. Interestingly, the change in ICP does not seem to be influenced by the dose administered or the route of administration. More data would be needed to analyze whether this reduced variation can be attributed to the possible reduction in CPP induced by DCF itself or to other factors. It would be interesting to assess and differentiate the real impact of DCF on MAP, CPP and ICP as a function of the preservation of patients’ cerebral autoregulation by Prx.

Side effects were poorly screened as they were monitored solely during the administration in four out of six studies. However, from the results reported by the studies that carried out the monitoring, we observed an absence of GI, renal or hepatic side effects. A possible explanation may be the fact that in these studies, the dose of DCF administered is low and the mode of administration is continuous perfusion; indeed, it is well known that an increase in complications is observed when DCF exceeds 150 mg/day and in repeated administrations [17]. Nevertheless, there is a lack of information on any gastric protection strategies implemented in those studies, which could mask a possible increase in GI effects. In the end, in light of its good antipyretic action, a low-dose diclofenac sodium (DCF) may be suitable for correcting fever and maintaining normothermia, with fewer side effects than full doses of anti-inflammatory drugs administered as an IV push [29].

Fears of facilitating development or worsening infections cannot be eliminated as most of the patients were administered antibiotics, although no changes or increase in inflammatory parameters measured during the administration period were reported. Moreover, although most of the origins of fever in the studies are infectious and only a few patients are identified as presenting central-order fever, no difference in the effect of DCF was detected in the two types of fever. No conclusions can be drawn from such studies. Reports have suggested that neurogenic fevers are somewhat resistant to traditional pharmacologic therapies [30,31]. One study showed that only 7% of patients with traumatic brain injury and 11% of patients with SAH defervesced with the antipyretics used [29]. In conclusion, the literature is poor when it comes to the impact of NSAIDs on the different types of fever in neuro-ICU.

Neurological outcomes were assessed in two studies using two different scales (Modified Rankin scale and Glasgow Outcome Score scale) at two different times, at 3 months and 6 months, respectively. Moreover, the authors only mentioned the absence of differences between groups. No conclusion on the use of DCF to improve the long-term neurological outcome can be made because of heterogeneity of these studies and the different way of assessing it. Although the long-term neurological outcome remains the main purpose of the early management of acute brain injury in neuro-ICU.

This systematic review provides an overview of the available literature on the use of diclofenac for fever control in critical care. We note that DCF has a good antipyretic effect but with a possible concomitant impact on MAP and thus on CPP. However, this review only provides IV° evidence. All but one of the studies included in this analysis were not randomized. All articles included in this review were peer-reviewed, but there was a possibility of publication bias as shown by the New Castel scale, and the heterogeneity in the studies reduces the strength of the available evidence. The scarcity of studies and data as well as the difference in dosage and route of administration of DCF appear to be an important limitation in the analysis of the results. In addition, the different site of temperature measurement between the different studies constitutes an additional risk of bias and error. In conclusion, despite the wide DCF use, only a few studies have been conducted so far and the evidence that can be extrapolated is scarce.

## 5. Conclusions

The findings of our systematic review confirm that diclofenac sodium is effective in reducing body temperature in patients with brain injury. The reduction in hemodynamic parameters (MAP and CPP) seems, however, more related to dose and route of administration of DCF. The use of DCF in a fashion of low dose and continuous intravenous administration appears to have less hemodynamic effects. Despite the fact that the use of DCF has been described since 1990’s in neuro-ICU, data in the literature are scarce and further studies are needed to evaluate the benefits of DCF in temperature control, its best dose and route of administration and the effects on brain parameters and long-term neurological outcome.

## Figures and Tables

**Table 1:** Newcastle–Ottawa Scale and Rob2 tool for risk of bias assessment for the included studies in this meta-analysis.

**Study**	**Study Design**	**Bias**									**Overall Bias**
		**Randomization Process**		**Deviation from Intended Interventions**		**Missing Outcome Data**	**Measurement of the Outcome**		**Selection of Reported Results**		
Cormio 2007 [21]	Prospective, RCT	Low risk		Some Concerns		Low Risk	Some Concerns		Some Concerns		Some Concerns
**Study**	**Study Design**	**Selection**				**Comparability of Cohorts**		**Outcome**			**NOS**
		**Representat-iveness of Sample**	**Non Exposed Cohort**	**Ascertain-ement**	**Outcome Not Present at Start**			**Asses-sment**	**Time of Follow-Up**	**Adequacy of Follow-Up**	
						**Main factor**	**Addition-nal Factor**				
Cormio 2000 [22]	Retrospective	*	/	*	*	*	*	/	/	/	5/9
Caricato 2004 [23]	Prospective	*	/	*	*	*	*	/	/	/	5/9
Cormio 2004 [24]	Prospective	*	/	*	*	*	*	/	/	/	5/9
Schiefeker 2013 [25]	Retrospective	*	/	*	*	*	*	/	*	*	7/9
Picetti 2016 [26]	Prospective	*	/	*	/	*	*	/	/	/	4/9

Risk of bias using NOS is shown in the bottom part of the table and evaluates risk of bias in the five observational studies; risk of bias assessed with the RoB2 tool for randomized control trial (RCT) is shown in upper part of the table. * Star for NOS risk of bias.

**Table 2:** Characteristics of included studies.

Study, Year	Study Design	# Patients/# Interventions	Outcome	Types of Neuro Injury	Control Group	Way of Administration	Dose of Diclofenac	Follow-Up	Adverse Events
Cormio 2000 [22]	Retrospective	12	Efficacy of continuous infusion in fever management, D hemodynamic and cerebral effects, AE	7 TBI, 5 SAH	No	Continuous intravenous infusion	0.02–0.08 mg/kg/h	No	Monitored during administration
Caricato 2004 [23]	Prospective	18	Efficacy of continuous infusion in fever management, hemodynamic and cerebral effects of D	Brain injuries (no specific details)	No	Continuous intravenous infusion	0.04 mg/kg/h	No	Monitored during administration
Cormio 2004 [24]	Prospective	35	Efficacy of continuous infusion in fever management, D hemodynamic and cerebral effects, AE	22 TBI, 7 SAH, 6 post op	No	Subcutaneous injection	0.17 mg/kg	No	Monitored during administration
Cormio 2007 [21]	Prospective, randomized, controlled clinical trial	22	Efficacy of continuous infusion versus intermittent bolus in fever management, D hemodynamic and cerebral effects, AE	12 TBI, 10 SAH	No	Continuous intravenous infusion versus intermittent bolus	0.04–0.08 mg/kg/h or intermittent bolus of 0.2 mg/kg Diclofenac; 10 mg Ketoprofene or 1000 mg Proparacetamol	No difference between 2 groups, Glasgow Outcome Scale	Monitored during administration
Schiefecker 2013 [25]	Retrospective	29/122	Effects of D on cerebral parameters	SAH	No	Intravenous infusion	75 mg	Modified Rankin Score at 3 months	Not monitored
Picetti 2016 [26]	Prospective	30	Effect of IM infusion on fever management	15 SAH, 6 TBI, 8 ICH, 1 stroke	No	Intramuscular injection	12.5 mg	No	Not monitored

Legends: AE = Adverse Events, IM = Intramuscular, D = Diclofenac (versus DCF), TBI = Trauma Brain Injury, SAH = Subarachnoid Hemorrhage, ICH = Intracranial Hemorrhage.

## Data Availability

No new data were created.

## Supplementary Materials

The following supporting information can be downloaded at: https://www.mdpi.com/article/10.3390/jcm12103443/s1, Study flow chart; Research strategy; PRISMA checklist. Table S1. Effects of Diclofenac administration on body temperature and cerebral parameters.
