# Supplementary material for: Diclofenac Sodium for Fever Control in Neurocritical Care: A Systematic Review
Source: J Clin Med. 2023 May 13;12(10):3443. doi: 10.3390/jcm12103443 (PMC10219558; doi:10.3390/jcm12103443)
Supplement: Supplementary file 1 [file jcm-12-03443-s001.zip › Supplementary files/Detailed search strategies.pdf]

Appendix S1

Detailed search strategies.
Search strategies for the article appendix:

Medline:

("diclofenac sodium"[Mesh] OR "diclofenac"[Mesh] OR "diclofenac sodium"[tiab] OR "voltaren"[tiab] OR “Anti-Inflammatory Agents"[Mesh] OR "anti-inflammatory agents [tiab]) AND ("Fever"[Mesh] OR “temperature”[Mesh] OR "fever"[tiab]) AND (“critical care”[Mesh] OR "intensive care unit"[Mesh] OR "intensive"[tiab] OR “neurocritical care”[tiab] OR "ICU"[tiab]) NOT (exp CHILD/ not exp ADULT/, exp Animals/ not Humans/, case reports)

Cochrane library ® search in the search engine with Keyword or Title abstract keyword :

(diclofenac sodium* or diclofenac* or voltaren* or sodium).ab,hw,ti. AND (acute or intensive care or intensive or ((critical or neurocritical) adj2 care)).ab,hw,ti. AND (fever or temperature).ab,hw,ti.

Scopus via Elsevier:

((TITLE-ABS-KEY (diclofenac* OR sodium OR diclofenac sodium * OR Anti-Inflammatory Agents *) AND TITLE-ABS-KEY (fever* OR temperature* OR body temperature) AND TITLE-ABS-KEY(intensive care* OR critical* OR care* OR icu* OR neurocritical care) AND NOT ((TITLE (case-report*))
