# Supplementary material for: Diclofenac Sodium for Fever Control in Neurocritical Care: A Systematic Review
Source: J Clin Med. 2023 May 13;12(10):3443. doi: 10.3390/jcm12103443 (PMC10219558; doi:10.3390/jcm12103443)
Supplement: Supplementary file 1 [file jcm-12-03443-s001.zip › Supplementary files/Study Flow .pdf]

Figure . Study Flow

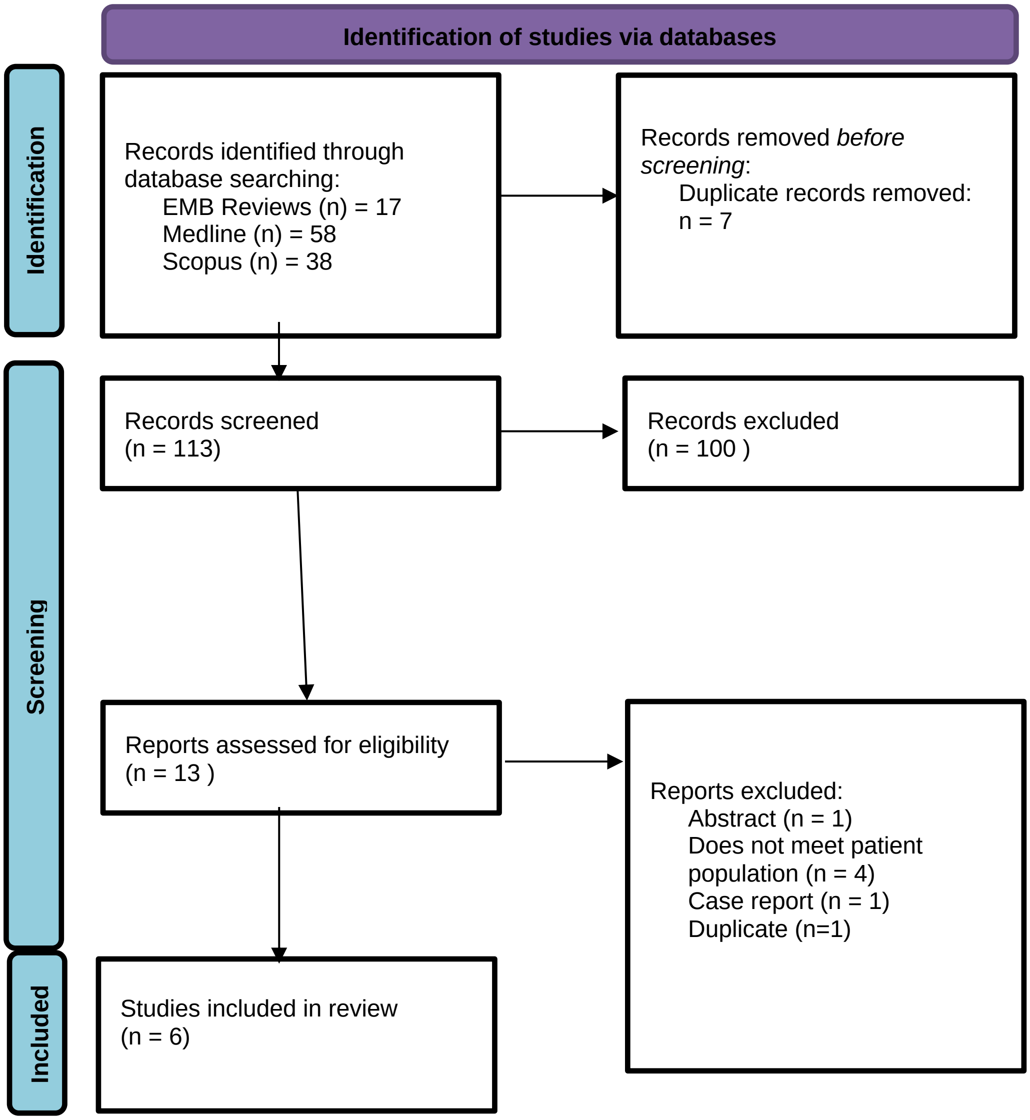

Preferred Reporting Items for Systematic Reviews and Meta-analyses Study Flow
