# Supplementary material for: Diclofenac Sodium for Fever Control in Neurocritical Care: A Systematic Review
Source: J Clin Med. 2023 May 13;12(10):3443. doi: 10.3390/jcm12103443 (PMC10219558; doi:10.3390/jcm12103443)
Supplement: Supplementary file 1 [file jcm-12-03443-s001.zip › Supplementary files/Tables.pdf]

Table S1 Effects of Diclofenac administration on body temperature and cerebral parameters

Study, year	Day of study	GCS before (mean)	T° type of measure (°C)	T° before	T° after	Time of administration	MAP	HR	CPP (mmHg)	CCP before	CCP after	ICP (mmHg)	ICP before	ICP after
Cormio 2000	4,5	7 ± 2	Axillary	38,5 ± 0,4	36,9 ± 0,5	48 hours	Stable	Normalised	Slight increase	75,3 ± 11,8	89,1 ± 12,8	Decreased	22,7 ± 6,6	12,1 ± 6,6
Caricato 2004	0,16	≤ 7	Bladder	38,6 ± 0,1	36,8 ± 0,3	24h at least	Slight decrease after 6 hours	Stable	Stable	No data	No data	Slight decrease	No data	No data
Cormio 2004	7	5	No data	38,4 ± 0,4	37,6 ± 0,5	Repeated doses	Slight decrease	Decrease	Stable	71 ± 15	69 ± 15	Decreased	16 ± 8	12,6 ± 6
Cormio 2007	2,6	≤ 8	Bladder	38,3	37,3 ± 0,2	6,6 ± 2,2 days	Increased	Not monitored	Increased	71	79 ± 16	Decreased	17	15,7 ± 6,6
Schiefecker 2013	No data	No data	Bladder	38,3 ± 0,05	37,5 ± 0,05	No data	Decreased	Not monitored	Slight decrease	85 ± 1,4	76 (no interval)	No change	8,6 ± 0,4	No change
Picetti 2016	No data	≤ 8	Bladder / pulmonary artery catheter	37,8 ± 0,3	37,5 ± 0,5	2 hours	Slight decrease	Slight decrease	Slight decrease	86,8 ± 14	74,1 ± 11	No change	15,5 ± 5,1	15,4 ± 4,6

Legends : T° = Body temperature; MAP = mean arterial pressure: HR = heart rate; CPP = cerebral pulsed pressure, ICP = intracranial pressure
